# Effective components of feedback from Routine Outcome Monitoring (ROM) in youth mental health care: study protocol of a three-arm parallel-group randomized controlled trial

**DOI:** 10.1186/1471-244X-14-3

**Published:** 2014-01-06

**Authors:** Maartje AMS van Sonsbeek, Giel GJM Hutschemaekers, Jan Willem Veerman, Bea BG Tiemens

**Affiliations:** 1 Behavioural Science Institute, Radboud University Nijmegen, Montessorilaan 3, 6525 HR Nijmegen, The Netherlands; 2 Pro Persona Centre for Education & Science (ProCES), Tarweweg 6, 6534 AM Nijmegen, The Netherlands; 3 Pro Persona Youth Tiel, Siependaallaan 3, 4003 LE Tiel, The Netherlands

**Keywords:** Routine Outcome Monitoring, Feedback, Youth mental health care, Randomized controlled trial

## Abstract

**Background:**

Routine Outcome Monitoring refers to regular measurements of clients’ progress in clinical practice, aiming to evaluate and, if necessary, adapt treatment. Clients fill out questionnaires and clinicians receive feedback about the results. Studies concerning feedback in youth mental health care are rare. The effects of feedback, the importance of specific aspects of feedback, and the mechanisms underlying the effects of feedback are unknown. In the present study, several potentially effective components of feedback from Routine Outcome Monitoring in youth mental health care in the Netherlands are investigated.

**Methods/Design:**

We will examine three different forms of feedback through a three-arm parallel-group randomized controlled trial. 432 children and adolescents (aged 4 to 17 years) and their parents, who have been referred to mental health care institution Pro Persona, will be randomly assigned to one of three feedback conditions (144 participants per condition). Randomization will be stratified by age of the child or adolescent and by department. All participants fill out questionnaires at the start of treatment, one and a half months after the start of treatment, every three months during treatment, and at the end of treatment. Participants in the second and third feedback conditions fill out an additional questionnaire. In condition 1, clinicians receive basic feedback regarding clients’ symptoms and quality of life. In condition 2, the feedback of condition 1 is extended with feedback regarding possible obstacles to a good outcome and with practical suggestions. In condition 3, the feedback of condition 2 is discussed with a colleague while following a standardized format for case consultation. The primary outcome measure is symptom severity and secondary outcome measures are quality of life, satisfaction with treatment, number of sessions, length of treatment, and rates of dropout. We will also examine the role of being not on track (not responding to treatment).

**Discussion:**

This study contributes to the identification of effective components of feedback and a better understanding of how feedback functions in real-world clinical practice. If the different feedback components prove to be effective, this can help to support and improve the care for youth.

**Trial registration:**

Dutch Trial Register NTR4234

## Background

Each year about 270.000 children, adolescents and parents/caregivers^a^ are referred to youth departments of mental health care institutions in the Netherlands [1]. The success rates of treatments at these institutions (mainly psychotherapy) are low. The small body of outcome studies in community-based, usual care settings in the United States has yielded a mean effect size near zero [2] and estimates of deterioration between 14% and 24% [3]. Furthermore, it is estimated that 40% to 60% of the children and adolescents discontinue treatment prematurely (dropout). Many of these dropouts are likely due to a perceived lack of benefit from treatment [4].

Clients who experience no change or negative change will require a disproportionately greater amount of treatment resources. Prevention of such treatment failures, therefore, becomes an important goal of clinical practice. In addition, clinicians are seldom capable of predicting which clients will and will not improve through treatment [5]. Factors that might be related to clients’ deterioration include symptom increase, ruptures in the working alliance and treatment goal failure. Although clinicians might recognize these factors, they have trouble noticing these cues in their clients [6]. Clinicians need more systematic and reliable information about the status of their clients [7]. Consequently, there has been a growing awareness in research and practice that clients’ response to treatment should be evaluated in a more systematic and structured way in order to adjust treatment when clients do not respond according to expectation. Doing so would likely improve treatment outcomes [8].

Routine Outcome Monitoring (ROM) refers to regular measurements of clients’ progress in clinical practice [9], using standardized instruments, aiming to evaluate and, if necessary, adapt treatment [10]. Clients are invited to fill out questionnaires at the beginning of treatment, during treatment and at the end of treatment. Subsequently, clinicians are provided with feedback about their clients’ response to treatment [11]. Based on the feedback, clinicians can make decisions on continuing, altering or terminating treatment [12]. Thus, feedback focuses on the gap between the desired and actual results of care, and possible associated factors, that are within the control of the health care provider [13]. Therefore, feedback interventions are a promising approach to improve clinical practice [7].

### Effects of feedback

Comprehensive and repeatedly updated reviews in general healthcare have shown that audit and feedback can have small to moderate effects on healthcare professionals’ compliance with desired practice and on client outcomes [14]. However, effects vary according to the way the intervention is designed and delivered. In adult mental health care, research on the effects of feedback has shown slightly negative to large positive effects [15-17]. Positive effects are shown on the accuracy of diagnosis [18,19] and on communication between the client and clinician [16,20]. Feedback can also significantly enhance treatment results [21], even doubling the overall effect size of treatment [22], and it can decrease the number of treatment sessions [23]. Moreover, treatment results in feedback groups are maintained for a much longer time [24] and less treatments are failing when clinicians receive feedback [25]. Nevertheless, more than one-third of the feedback interventions have been shown to decrease performance [26] and the effects of feedback did not prevail in the long term [16].

Studies that have specifically investigated the effects of feedback in youth mental health care are rare. To the best of our knowledge, Bickman et al. [27] is the only such published study, concerning home-based mental health treatment for children and adolescents in community settings. Assessments by children, adolescents, parents and clinicians indicated that children and adolescents whose clinician had access to weekly feedback improved faster than children and adolescents whose clinician received quarterly feedback. Additionally, a dose–response analysis showed that effects were stronger when clinicians viewed more feedback reports.

### Evidence for feedback mechanisms

Most theories (e.g. Contextualized Feedback Intervention Theory [28]) start with a comparison between the content of the feedback (in mental health care the client’s current health status and progress in treatment so far) and a goal (recovery). This comparison creates a positive or a negative evaluation of the clinician’s performance.

Clinicians who are dissatisfied with their performance will change their behaviour if they are committed to the goal and if they meet a threshold level of self-efficacy for the task (cf. Goal-Setting Theory [29]). This mechanism is in accordance with studies in general healthcare, which show that feedback is more effective when clinicians previously agreed to review their practice [30] and when they are actively involved and have specific and formal responsibilities for implementing change [31]. Theory further states that detailed plans regarding where, when and how behaviours will be enacted may increase goal-directed behaviours by increasing self-efficacy [13]. Additionally, action plans can facilitate success by increasing goal-commitment. These mechanisms have also been confirmed in general healthcare by research showing that feedback is more effective when it is more concrete; given more frequently (at least once a month) and both verbal and written, presented close to the time of decision-making, aiming to decrease rather than increase provider behaviours, and offering instructions with both explicit goals and a specific action plan [14]. In mental health care, research has suggested that feedback should (a) be delivered as promptly as possible after the data have been collected to allow clinicians to perceive the connection between the feedback and their behaviour, (b) be given frequently so that changes in processes and outcomes can be observed as they occur and corrective actions can be applied if necessary, (c) be specific, (d) be written or graphic instead of verbally delivered, (e) should focus on the clinician’s actual behaviour instead of clinical outcomes, (f) be supplemented with a directive intervention and concrete suggestions about ways to improve and (g) be tailored to the needs and preferences of the recipients [21,26,32,33].

Alternatively, feedback may be perceived as inaccurate and therefore disregarded, resulting in external attribution of reasons for a lack of progress [34], reduction in commitment to the goal [21] or the recipient lowering the goal to make it more achievable (cf. control theory [35]). This mechanism has been directly or indirectly confirmed in studies showing that if clinicians do not consider the feedback credible, valid, informative, or useful, they are more likely to dismiss it whenever it does not fit their own preferences [36]. It has also been shown that characteristics of clinicians, namely whether they have an internal or an external feedback propensity, are committed to using the feedback and are self-efficacious, can moderate the effects of feedback [37]. Additionally, there can be barriers for clinicians to use feedback, such as no accountability for using feedback and organizational factors [28] like high administrative pressures, a heavy caseload, lack of time, having other tasks that are competing for attention and having difficulty interpreting the feedback [37].

Clients can benefit from feedback only when clinicians pay attention to and use feedback [38]. However, feedback may not be effective under all circumstances [39]. Repeatedly, researchers have found that feedback appears to be most effective for clients who are not progressing well in treatment or who are predicted to have a poor treatment response, i.e. the not on track (NOT) cases [9,17,40]. The largest treatment gains for potential non-responders are achieved when feedback is supplemented with clinical support tools. These tools are proposed as an evidence-based problem-solving strategy and are arranged in a decision tree to direct clinicians’ attention to certain factors known to be important in treatment outcome [32].

### Aims and hypotheses

ROM has become an increasingly popular method for improving treatment and has been adopted by many mental health care institutions worldwide [22,41,42]. Although it seems that feedback from ROM has potential to enhance outcomes in clinical practice, there are still many unanswered questions. There is a need to operationalize and directly compare different approaches to improve the design and delivery of feedback. Also, in order to explain why feedback leads to improvement in some cases but not in others, more insight is needed into the processes underlying feedback. Moreover, barely research has been conducted in real-world clinical practice and outside the United States. Studies that have specifically investigated the effects of feedback in youth mental health care are rare.

The aim of this study is to investigate several potentially effective components of feedback from ROM in youth mental health care in the Netherlands. We will compare three different feedback conditions. In all three conditions feedback will be given to clinicians, will consist of written and graphic performance results, will be compared to norms, will be given at the start of treatment, one and a half months after the start of treatment and every three months during treatment and will be delivered one day after data collection.

In the first feedback condition, clinicians will receive basic feedback regarding clients’ symptoms and quality of life. However, reduction of symptoms or enhancement of quality of life is not always the primary or the single goal of treatment [43]. Also, children’s and adolescents’ symptoms and problems often arise in interaction with family members [44]. Furthermore, many children and adolescents have chronic health problems (e.g. ADHD or ASD), which might well persist into adulthood [45,46]. Frequently, children, adolescents and their parents want to learn how to cope with their problems and overcome obstacles that they encounter. Hence, it seems important to assess aspects like self-efficacy, social network and parenting skills, include the results in the feedback to the clinicians and provide clinicians with possible solutions if clients are not responding to treatment. This strategy is consistent with theory and research suggesting that more concrete and practical feedback is more effective. Thus, in the second feedback condition, we will enhance the feedback of the first condition by including information about possible obstacles to a good outcome that clients may be facing (e.g. problems with self-efficacy and the social network but also motivation and the therapeutic alliance) and practical suggestions for improving treatment (youth clinical support tools). Therefore, the feedback will be more specific than in the first condition, will be more tailored to the needs of the clinicians and will provide more information about the clinicians’ actual behaviour.

Although feedback might be intended to be practical and useful, literature shows that if feedback is regarded as inaccurate, incredible or invalid, clinicians might reject the feedback, attribute a lack of progress to external reasons and lower the goal of treatment. Accordingly, in the third feedback condition, we will prevent clinicians from disregarding the feedback. In this condition, the feedback that is provided in the second condition will be discussed with a colleague using a standardized format for case consultation. Because peers who have personal relevance for the clinician will give additional advice about ways to improve treatment, clinicians will probably consider the feedback as more credible, valid, informative and useful and will be more likely to accept the feedback. Therefore, compared to the other conditions, clinicians in the third feedback condition will pay more attention to the feedback, will become more involved and might feel more responsible to act upon the feedback.

The primary outcome measure will be symptom severity and secondary outcome measures will be quality of life, satisfaction with treatment, number of sessions, length of treatment and rate of dropout. Compared to the first condition, we expect that in the second and third feedback conditions (a) children’s and adolescents’ symptoms will decrease more rapidly and to a greater extent, (b) children’s and adolescents’ quality of life will improve more, (c) children, adolescents and parents will be more satisfied at the end of treatment, (d) treatments will contain fewer sessions, (e) treatments will be shorter, and (f) children, adolescents and parents will drop out of treatment less often. Overall, we expect that the largest effects of feedback will occur in the third feedback condition, where the basic feedback will be enhanced with additional information and practical suggestions, and clinicians will discuss the feedback in case consultation with a colleague. Additionally, we expect that feedback will be most effective for children, adolescents and parents who are not progressing well in treatment (i.e. are NOT).

## Methods/Design

### Trial design

**Figure 1:**
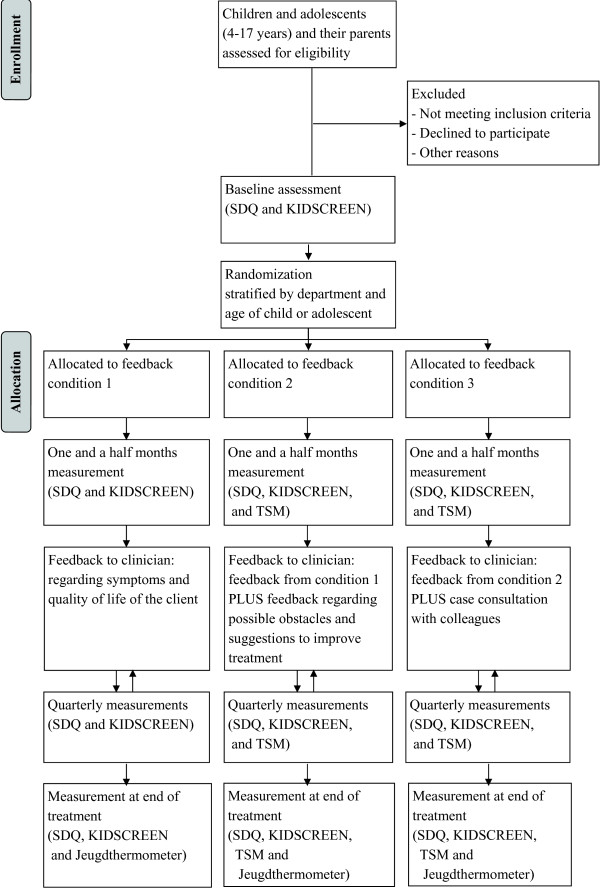
Study design.

We will examine three different forms of feedback from ROM in youth mental health care through a three-arm parallel-group randomized controlled trial (see Figure 1 for the study design). The first condition (control condition) will include basic feedback about the client’s symptoms and quality of life. The second condition will include the same feedback as the first condition, but the feedback will be enhanced with information regarding possible obstacles to a good outcome that the client may be facing and with practical suggestions to improve treatment (youth clinical support tools). The third condition will include the same feedback as the second condition, but in addition the clinician will discuss the feedback with a colleague using a standardized format for case consultation.

The trial will be conducted at mental health care institution Pro Persona. All outpatient youth departments (in Arnhem, Ede, Nijmegen and Tiel) will participate and all families with children and adolescents between the ages of 4 and 17 years will be approached to participate. If the child is between 4 and 11 years old, the parents will be approached to participate in the study. If the child or adolescent is between 12 and 17 years old, the child or adolescent will be approached to participate in the study. Children under 16 years of age will only be approached to participate after parental consent for participation in the study is obtained. Children, adolescents and parents who agree to participate will sign an informed consent form. The participants will be randomly allocated to one of the three feedback conditions (144 participants per condition; randomization ratio [1:1:1]). All participants will fill out web-based questionnaires and their clinicians will receive feedback about the results. Clinicians will have to discuss the feedback with the participants during treatment. Participants will not receive incentives for completing the questionnaires, as this trial is part of the regular ROM within the institution. Ethical approval has been granted by the ethical committee of the Faculty of Social Sciences at the Radboud University Nijmegen (ECG 2012-1304-031).

### Participants

The study will take place in all of Pro Persona’s outpatient youth departments (in Arnhem, Ede, Nijmegen and Tiel). Pro Persona is a specialised mental health care institution in the Netherlands that offers outpatient and inpatient treatment for children, adolescents, adults and the elderly. Approximately 110 clinicians work in the outpatient youth departments. These clinicians are social workers, social psychiatric nurses, nurse practitioners, educationists, psychologists, health care psychologists (GZ-psychologen), clinical psychologists, psychiatrists and physicians.

All families with children and adolescents between the ages of 4 and 17 years who are referred to Pro Persona’s outpatient youth departments receive information about the study. We will include children and adolescents with any kind of mental health problems (e.g. developmental, anxiety or mood disorders) and any kind of treatment (e.g. individual or group treatment, cognitive-behavioural or solution-focused treatment, frequent or irregular treatment). On average, 33% of the treatments in youth mental health care last less than 3 months, 35% last from 3 to 12 months and 31% last more than a year [47]. The only exclusion criterion will be child’s, adolescent’s or parent’s insufficient understanding of the Dutch language. If the child is between 4 and 11 years old, the parents will be approached to participate in the study, because younger children are less able to fill out questionnaires and parents are legally responsible for the children. If the child or adolescent is between 12 and 17 years old, the child or adolescent will be approached to participate in the study. Children and adolescents under 16 years of age will only be approached to participate after parental consent for participation in the study is obtained. Children, adolescents and parents who agree to participate will sign an informed consent form. Participants will be randomly allocated to one of the three feedback conditions (144 participants per condition), using a block randomization scheme. Randomization will be carried out by an independent researcher after the baseline assessment.

We are aware that prior research has shown systematic discrepancies between multiple informants with regard to the nature and severity of children’s and adolescents’ symptoms [48,49]. However, youth departments of mental health care institutions treat children and adolescents of all ages and we would like to generalize the results of this study to all age groups. To ensure that the distribution of parents and children and adolescents is similar in the three feedback conditions, randomization will be stratified by age of the child or adolescent (4 to 11 years old and 12 to 17 years old).

### Procedure

Depending on the age of the child or adolescent and the informed consent form, we will invite the child or adolescent or the parents to fill out web-based questionnaires. If children are between 4 and 11 years old, the parents will be invited if they have signed the informed consent form. If children and adolescents are between 12 and 17 years old, the children or adolescents will be invited if they have signed the informed consent form. Children and adolescents under 16 years of age will only be invited after parental consent for participation in the study is obtained. In accordance with the preferences and capabilities of Pro Persona, the children, adolescents and parents will be invited to fill out questionnaires at the start of treatment (baseline assessment), one and a half months after the start of treatment, every three months during treatment and at the end of treatment. Therefore, one-third of the children, adolescents and parents will have one measurement during treatment, one-third will have four measurements during treatment, and one-third will have at least five measurements during treatment.

In the first feedback condition, children, adolescents and parents will fill out only the standard set of questionnaires. These questionnaires measure client’s symptoms (SDQ) and quality of life (KIDSCREEN) and satisfaction with treatment (only at the end of treatment; Jeugdthermometer). In the second and third feedback conditions, children, adolescents and parents will fill out an additional questionnaire (TSM-Y or TSM-P), which measures possible obstacles to a good outcome that they may be facing during treatment. Filling out the questionnaires takes between 30 minutes (standard set) and 45 minutes (maximum set of questionnaires and the satisfaction questionnaire). Children, adolescents and parents will be invited to fill out the questionnaires via an e-mail message that includes a link to a fully computerized ROM-system (NetQ-ROM). After children, adolescents and parents have completed the questionnaires, the system will score the questionnaires and generate feedback reports (in the form of PDF-files). Each night, the system will incorporate new feedback reports into the electronic patient file. Subsequently, the clinician will discuss the feedback with the client to determine whether and how treatment needs to be modified. The data that are gathered in the system can be exported to an SPSS or an Excel file at any time.

### Intervention

The trial will include three different feedback conditions: basic feedback regarding the client’s symptoms and quality of life (control condition), feedback from the first condition enhanced with information regarding possible obstacles to a good outcome that the client may be facing and with practical suggestions to improve treatment (youth clinical support tools), and feedback from the second condition which the clinician will discuss with a colleague using a standardized format for case consultation.

In the first condition, the feedback will include tables and graphs with the results from the standard set of questionnaires (total scores and subscale scores), except for the results from the satisfaction questionnaire (administered only at the end of treatment). The tables and graphs will show (a) the range in which children’s, adolescents’ or parents’ scores fall compared to norms (e.g. normal, subclinical, clinical), (b) changes in the scores across the measurements, and (c) items which are considered to be critical and in need of special attention. Additional file 1 provides an example of the different parts of this feedback report.

In the second and third feedback conditions, children, adolescents and parents will also fill out the Treatment Support Measure–Youth Form (TSM-Y; 40 items) or the Treatment Support Measure–Parent Form (TSM-P; 40 items) [50]. The TSM-Y and TSM-P domains and items were selected because of their association with positive treatment outcomes in longitudinal research with 750 children, adolescents and their parents served in community mental health systems [51] and their importance in the literature on youth psychotherapy outcome [52]. For children and adolescents, the domains are: (1) feelings of self-efficacy, (2) supportive relationships with family members and friends, (3) the child’s or adolescent’s motivation to participate in treatment, and (4) the alliance between the child or adolescent and the clinician. For parents, the domains are: (1) the parent’s sense of confidence in performing various parenting tasks (parenting self-efficacy), (2) the parent’s own network of supportive relationships, (3) specific parenting skills and behaviours, (4) parental distress (including personal problems and stress related to parenting) and (5) the alliance between the parent and the therapist of the child or adolescent. We will also ask about the alliance between the parent and his or her own therapist. The feedback in the second and third conditions will include tables and graphs with results both from the standard set of questionnaires (total scores and subscale scores) and the TSM-Y or TSM-P (subscale scores). The tables and graphs related to the TSM-Y or TSM-P show (a) in what range the children’s, adolescents’ or parents’ scores fall compared to norms (e.g. below or above the cut-off scores), (b) changes in the scores across the measurements and (c) items which are considered critical and in need of special attention. If children, adolescents or parents fall above the cut-off score for one or more domains, practical suggestions are given for interventions to be considered by the clinician (youth clinical support tools; e.g. structure activities in treatment and homework assignments to provide opportunities for the child or adolescent to experience success in targeted areas; encourage the child or adolescent to envision and describe how he/she wants his/her life to be; role play social situations to facilitate acquisition of social skills; give and ask for feedback on the therapeutic relationship). By discussing the feedback, the clinician and child, adolescent or parent can adjust treatment together, in order to get back on track for a good outcome. The assumption is that clinicians intend to provide the best care, but are uncertain about how to change their behaviour if the treatment is not working. Feedback can facilitate actions to adapt treatment.

In addition to the procedure in the second feedback condition, clinicians in the third feedback condition will discuss the feedback with a colleague. In order to structure the discussion, clinicians will be given a standardized format for case consultation. They will be required to complete the format and return it to the researcher. Thus, clinicians will be obliged to pay attention to the feedback and will receive additional advice from their colleagues about ways to improve treatment.

In all of the conditions, we will monitor whether clinicians actually discuss the feedback with their clients by asking children, adolescents and parents within the standard set of questionnaires. In the third feedback condition, we will also monitor whether clinicians actually discuss the feedback with a colleague. After all, if clinicians do not use feedback constructively, it is unlikely that outcomes will improve.

Before the start of the trial, all clinicians of the participating youth departments will be trained to interpret the feedback, discuss the feedback with clients and use the standardized format for case consultation with a colleague. Besides that, a ROM-implementation package with various kinds of tools has been developed to help clinicians with ROM and use of the feedback. In addition, a helpdesk will be available for children, adolescents, parents and clinicians.

### Outcome measures

#### Primary outcome measure

The primary outcome measure will be symptom severity, which will be measured with the Dutch version of the Strengths and Difficulties Questionnaire (SDQ [53,54]). The SDQ will be filled out by parents when the child is between 4 and 11 years old and by children or adolescents when they are between 12 and 17 years old. For the first assessment, the extended versions of the SDQ for both parents and children and adolescents will be used. Each version consists of 25 items on strengths and difficulties and an additional impact supplement. The 25 items are scored on a 3-point scale ranging from 0 (not true) to 2 (completely true). These items are divided into five subscales: emotional symptoms, conduct problems, hyperactivity-inattention, peer relationship problems and prosocial behaviour. A total difficulties score can also be calculated by summing the scores on the emotional symptoms, conduct problems, hyperactivity-inattention, and peer problems subscales (total score can range from 0 to 40). The impact supplement asks whether the respondent thinks the child or adolescents has a problem, and, if so, inquires further about chronicity, distress, social impairment, and burden to others. For the measurements during treatment, the follow-up versions of the SDQ for both parents and children and adolescents will be used. These versions include the 25 items on strengths and difficulties, the impact question and two additional follow-up questions (‘Has the intervention reduced problems?’ and ‘Has the intervention helped in other ways, e.g. making the problems more bearable?’). The parent- and self-report versions of the SDQ have shown acceptable internal consistency, test-retest stability, and parent-youth agreement and good concurrent validity in a Dutch community sample [54,55]. However, reliability seems insufficient for two subscales of the parent version – conduct problems and peer relationship problems [56]. Therefore, the total difficulties score of the parent- and self-report versions will be used in the analyses.

#### Secondary outcome measures

Secondary outcome measures will be quality of life, satisfaction with treatment, number of sessions, length of treatment and rate of dropout. Quality of life will be measured with the KIDSCREEN [57]. The KIDSCREEN-27 parent version will be used for parents of children between 4 and 11 years old and the KIDSCREEN-52 child-adolescent version will be used for children and adolescents between 12 and 17 years old. The items of the KIDSCREEN-27 and the KIDSCREEN-52 are scored on 5-point scales ranging from not at all/never to totally/always. The KIDSCREEN-27 consists of 27 items that can be divided into five subscales: psychical well-being, psychological well-being, autonomy and parent relation, social support and peers, and school environment. The KIDSCREEN-52 consists of 52 items that can be divided into ten subscales: physical well-being, psychological well-being, moods and emotions, self-perception, autonomy, parent relation and home life, financial resources, social support and peers, school environment, and social acceptance (vs. bullying). The KIDSCREEN-27 parent version has shown satisfactory item internal consistency, item discriminant validity and agreement between youth and proxy reports, and good reliability [57]. The KIDSCREEN-52 child-adolescent version has shown acceptable to satisfactory levels of reliability and validity [57,58].

Satisfaction with treatment will be measured with the Jeugdthermometer [59,60]. The parent versions that pertain to the treatment of the child and to the parenting skills training will be used for parents of children and adolescents between 4 and 11 years old. The child version will be used for children and adolescents between 12 and 17 years old. The three respective versions of the Jeugdthermometer consist of 31, 32 and 28 items that are either answered yes/no, ask for a rating or are open-ended. All versions ask for appraisal of information, appraisal of participation, appraisal of the clinician (the child’s clinician or the own clinician), appraisal of the treatment result and for background information. The child version of the Jeugdthermometer has shown acceptable to satisfactory reliability and the parent versions have shown good reliability [59]. The internal consistency of the parent version that pertains to the treatment of the child has not yet been clearly demonstrated, but the internal consistency of the parent version pertaining to the parenting skills training is good [59].

The number of treatment sessions will be counted (quantitative variable) and the length of treatment will be registered in days (quantitative variable) for each client. Dropout rates will be calculated as the percentage of clients that abandon treatment (registered as unilateral decision to end treatment) in each feedback group.

#### Moderator and potential covariates

In addition, we will examine whether being NOT moderates the association between the feedback intervention and symptom severity. We will determine whether each client is NOT by calculating the reliable change index (RCI [61]) for the total difficulties score on the SDQ. We will calculate the RCI by comparing the total difficulties score at the first measurement during treatment (one and a half months after treatment has begun) with the total difficulties score at the beginning of treatment (baseline assessment). Clients will be designated NOT if the RCI is smaller than 1.96 (i.e. not showing statistically significant improvement).

Lastly, socio-demographic characteristics of the children, adolescents and parents (e.g. sex, age, educational level, ethnicity, socioeconomic status, marital status and household composition) and clients’ diagnosis will be extracted from the electronic patient files and by asking additional questions.

### Sample size calculation

Literature indicates various effect sizes of feedback from ROM [17,21,27,62]. The present study was powered to detect the upper limit of a small effect size (f = 0.15; ANCOVA). A power analysis using G*Power [63] indicated that a total sample size of 432 (n = 144 per condition) would be sufficient to detect significant (α = .05, power = .80) condition effects on symptom severity (3 conditions, including testing for 3 covariates and possible interaction effects).

However, our previous experiences have shown that approximately 50% of the participants stop filling out questionnaires after one measurement (baseline assessment). Hence, the clinicians of these children, adolescents and parents do not receive feedback during treatment. It would not be desirable to impute 50% of the data as this would make the imputation highly unreliable. Therefore, we will invite twice as many children, adolescents and parents (n = 288 per condition and 864 in total) to include the required number of 144 participants per condition of whom clinicians have received feedback during treatment (i.e. at least 50% of the measurements).

### Randomization

Randomization of each participant to one of the three feedback conditions will be carried out using a computer-generated random number list with a blocked randomization scheme (block size 6, randomization ratio [1:1:1]). An independent researcher will allocate the participants to the feedback conditions after the baseline assessment. To ensure that the distribution of parents and children and adolescents is similar across the three feedback conditions, randomization will be stratified by age of the child or adolescent (4 to 11 years old and 12 to 17 years old). To exclude confounding due to differences among departments, randomization will also be stratified by department (Arnhem, Ede, Nijmegen and Tiel).

### Statistical analyses

All analyses will be conducted using Mplus [64], because this program allows analyses of complex data while taking into account the longitudinal character of the data. Intervention effects will be analyzed according to the intention-to-treat principle and the completers-only framework. For the intention-to-treat analysis, missing data will be handled using multiple imputations (MI). Descriptive statistics will be calculated for all variables of interest (e.g. symptoms, quality of life, satisfaction with treatment, number of sessions, length of treatment, dropout rate). In order to assess whether randomization resulted in equal groups, we will examine whether there are differences among the three feedback groups on relevant covariates (e.g. sex, age, educational level, ethnicity, socioeconomic status, marital status, household composition and diagnosis) using ANOVA for the continuous variables and Chi-Square tests for the categorical variables. Variables that are distributed differently among the three groups will be entered as control variables in all models testing the effects of the feedback conditions. The hypotheses will be tested using multivariate linear regression analyses. In addition, we will examine whether being NOT moderates the association between the feedback intervention and symptom severity. Therefore, interaction terms will be calculated as the product of the feedback condition and whether or not the client is NOT. These terms will be added as a second step in the multivariate analyses. Reporting of the results of the study will be in accordance with the CONSORT statement [65,66].

## Discussion

This study protocol describes a three-arm parallel-group randomized controlled trial to investigate several potentially effective components of feedback from ROM in youth mental health care in the Netherlands. It is hypothesized that the more specific and concrete the feedback, the more and faster children’s and adolescents’ symptoms will decrease, the more children’s and adolescents’ quality of life will improve and the more children, adolescents and parents will be satisfied at the end of treatment. In addition, we expect to find a negative relationship between feedback condition and the duration of treatment. It is hypothesized that the more specific and concrete the feedback, the fewer sessions treatments will contain and the shorter treatments will be. Also, we expect that children, adolescents and parents will drop out of treatment less often when feedback is more specific and concrete. We expect that the largest effects of feedback will occur when clinicians additionally discuss the feedback with a colleague. Finally, we expect that feedback will be most effective for children and adolescents who are not progressing well in treatment.

### Strengths and limitations

One of the strengths of this study is that we operationalize and directly compare three different forms of feedback to improve the design and delivery of feedback. The feedback conditions are derived directly from feedback theories and, to the best of our knowledge, the current study is the first to assess the effects of enhancing feedback with case consultation. Second, we focus not only on the effectiveness of different components of feedback, but also on the mechanisms through which feedback possibly works. We aim to understand why feedback is effective in some cases but not in others by testing whether not progressing well in treatment (i.e. not on track) moderates the effects of feedback. Third, we specifically investigate the effects of feedback in youth mental health care. We are thereby contributing to the limited knowledge about the effects of feedback and the importance of specific aspects of feedback for this particular population. Fourth, we actively involve parents in the study and combine self-reports with parent-reports. For children between 4 and 11 years old, parents fill out the questionnaires and clinicians will discuss the feedback with the parents. Hence, it is possible to assess the effects of different forms of feedback for young children and when only parents are receiving help (i.e. parenting skills training). Fifth, our study takes place in real-world clinical practice. The participating children, adolescents and parents are diverse in socio-demographic characteristics (e.g. sex, age, educational level, ethnicity, socioeconomic status, marital status and household composition) and the children and adolescents have all kinds of mental health problems. The study has only one exclusion criterion (insufficient understanding of the Dutch language). The results of the study will, therefore, have good external validity.

A limitation of the study is that differences in the care provided to participants in the second and third feedback conditions other than the intervention can arise (performance bias), because the intervention will be focused on the clinician. Since clinicians will not be blinded, it is possible that they will make additional efforts to improve treatment outcome when they receive feedback reports from the second and third feedback conditions instead of the first condition (control condition). Besides that, it is possible that effects from the third feedback condition will spill over to the second feedback condition. If clinicians have discussed several feedback reports with their colleagues (third feedback condition), they might guess what reactions their colleagues would have and what advice they would offer for clients who are not discussed (second feedback condition). Because each clinician will only have a couple of clients in each feedback condition, we think that such a spillover effect is unlikely. Furthermore, clients in the second and third feedback conditions may experience a placebo effect if their clinicians reveal that their feedback reports are being discussed with a colleague to improve treatment. In addition, due to technical and logistic restrictions, we are unable to check whether clinicians actually discuss the feedback with the children, adolescents and parents. However, we will remind the clinicians to discuss the feedback by sending them e-mail messages and we will ask clients at each measurement whether the feedback was discussed. Lastly, due to financial constraints, we will be unable to investigate the long-term effects of feedback in youth mental health care. Hence, further research should include follow-up measurements after treatment has ended.

### Implications for practice

By investigating several potentially effective components of feedback from ROM in youth mental health care, we contribute to the identification of important aspects of feedback and to a better understanding of possible mechanisms underlying feedback effects (e.g. what works for whom, when and how). We also contribute to the knowledge about and possibilities for use of feedback in the real world, through which the results of our study will be immediately relevant for clinical practice. Moreover, we extend the knowledge to youth mental health care in the Netherlands.

If the different components of feedback prove to be effective, the study will have strong practical relevance because the results will help to support the care for youth and to improve the treatments that children, adolescents and their parents receive. Clinicians might be better able to track the course of everyday treatments, signal potential treatment failures and adjust treatments accordingly by discussing the feedback with their clients. Eventually, treatments might have better outcomes, might contain fewer sessions and might become shorter. Also, children, adolescents and parents might drop out of treatment less often and might be more satisfied at the end of treatment. Consequently, there is potential for roll-out of the feedback with the most effective components across all youth departments of mental health care institutions throughout the Netherlands.

## Endnote

^a^Parents/caregivers might refer to any people who care for children and adolescents, e.g. biological parents, stepparents, adoptive parents, grandparents, other family members or guardians. Because children and adolescents are generally referred to mental health care with their biological parents or stepparents, we use the term ‘parents’ in the remainder of the study protocol.

## Abbreviations

ROM: Routine Outcome Monitoring; NOT: Not On Track; TSM-Y: Treatment Support Measure–Youth Form; TSM-P: Treatment Support Measure–Parent Form; SDQ: Strengths and Difficulties Questionnaire; RCI: reliable change index.

## Competing interests

The authors declare that they have no competing interests.

## Authors’ contributions

All authors contributed to the design of the study. MvS will be responsible for data collection, data analysis and for reporting the study results. GH, JWV and BT are supervisors and principal investigators. All authors read and approved the final manuscript.

## Pre-publication history

The pre-publication history for this paper can be accessed here:

http://www.biomedcentral.com/1471-244X/14/3/prepub

## Supplementary Material

Additional file 1 Example of a feedback report. Click here for file
