# Supplementary material for: Effective components of feedback from Routine Outcome Monitoring (ROM) in youth mental health care: study protocol of a three-arm parallel-group randomized controlled trial
Source: BMC Psychiatry. 2014 Jan 6;14:3. doi: 10.1186/1471-244X-14-3 (PMC3898381; doi:10.1186/1471-244X-14-3)
Supplement: Additional file 1 — Example of a feedback report. [file 1471-244X-14-3-S1.pdf]

Additional File 1: Example of a feedback report

K&J - jeugdigen SDQ en KIDSCREEN-52 (huidige scores op de SDQ)		
**SDQ-schaal**	**score**	**norm**
Emotionele problemen	2	normaal
Gedragsproblemen	5	klinisch
Hyperactiviteit/aandachtstekort	5	normaal
Problemen met leeftijdgenoten	5	klinisch
Prosociaal gedrag	5	subklinisch
Totaal	17	klinisch
Impactscore	0	normaal

K&J - jeugdigen SDQ en KIDSCREEN-52 (scoreverloop metingen SDQ)			
**SDQ-schaal**	**score Meting: 1**	**score Meting: 2**	**Vergelijking Meting: 2 - Meting: 1**
Emotionele problemen	2	2	0
Gedragsproblemen	10	5	-5
Hyperactiviteit/aandachtstekort	10	5	-5
Problemen met leeftijdgenoten	8	5	-3
Prosociaal gedrag	0	5	5
Impactscore	9	0	-9
Totaal	30	17	-13

K&J - jeugdigen SDQ en KIDSCREEN-52 (SDQ: subschaal problemen met leeftijdgenoten)

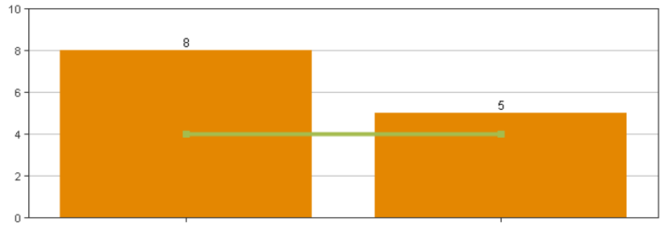

K&J - jeugdigen SDQ en KIDSCREEN-52 (huidige scores op de KIDSCREEN)		
**KIDSCREEN-schaal**	**score**	**norm**
Fysiek welzijn	15	lage score
Psychologisch welzijn	18	lage score
Stemming en emoties	28	gemiddelde score
Zelf-perceptie	16	lage score
Autonomie	20	gemiddelde score
Relatie met ouders en thuissituatie	24	gemiddelde score
Sociale steun en leeftijdgenoten	24	gemiddelde score
Schoolomgeving	24	gemiddelde score
Sociale acceptatie (pesten)	12	gemiddelde score
Financiële bronnen	15	hoge score

K&J - jeugdigen SDQ en KIDSCREEN-52 (KIDSCREEN: subschaal psychologisch welzijn)

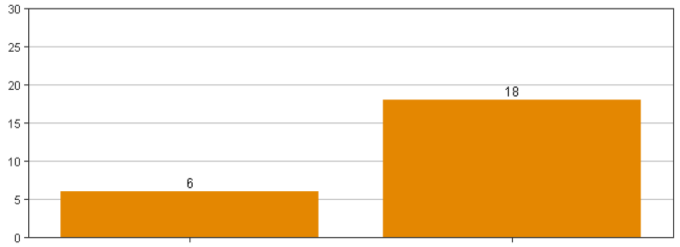

K&J - jeugdigen SDQ en KIDSCREEN-52 (kritieke items KIDSCREEN)			
**Item**	**Naam**	**Vraag**	**Antwoord**
KIDSCREEN-1.1	Lichamelijke activiteiten en gezondheid	Hoe is je gezondheid in het algemeen?	Slecht
KIDSCREEN-2.1	Gevoelens	Is je leven plezierig geweest?	helemaal niet
KIDSCREEN-3.1	Humeur	Heb je het gevoel gehad dat je alles verkeerd doet?	altijd
KIDSCREEN-6.1	Familie en thuis	Heb je het gevoel gehad dat je ouders je begrijpen?	helemaal niet
KIDSCREEN-6.2	Familie en thuis	Heb je het gevoel gehad dat je ouders van je houden?	helemaal niet
KIDSCREEN-6.3	Familie en thuis	Heb je je thuis gelukkig gevoeld?	nooit
KIDSCREEN-8.1	School en leren	Heb je het naar je zin gehad op school?	helemaal niet
